# Relationship between Asymmetry Profiles and Jump Performance in Youth Female Handball Players

**DOI:** 10.5114/jhk/163432

**Published:** 2023-07-15

**Authors:** Maria Cadens, Antoni Planas-Anzano, Xavier Peirau-Terés, Chris Bishop, Daniel Romero-Rodríguez, Marc Madruga-Parera

**Affiliations:** 1 Research Group into Human Movement, National Institute of Physical Education of Catalonia (INEFC), University of Lleida (UdL), Lleida, Spain.; 2 London Sport Institute, School of Science and Technology, Middlesex University, Greenlands Lane, London, UK.; 3 Physical Therapy Department, International University of Catalonia, Barcelona, Spain.; 4 INNOVITY, Barcelona, Spain.; 5 FC Barcelona Second Team, Sport Performance Area, Barcelona, Spain.; 6 reQ, Return to Play and Sports Training Center, Barcelona, Spain.

**Keywords:** unilateral, imbalances, youth, girl, team sport game

## Abstract

The relationships between asymmetries and performance or the identification of the asymmetry profile that has been routinely studied during jumping tests are largely derived from male-only and small sample size studies. Therefore, the aims of this study were: 1) to evaluate the magnitude and the direction of jump asymmetries (vertical and lateral) in three different age groups of adolescent female handball players, and 2) to identify the effect of asymmetry between limbs on jump performance in the three age groups. One hundred and eighty-five adolescent female handball players (age: 14.88 ± 1.49 years) were distributed in three groups: U14, U16 and U18 and performed two tests to assess jumping ability which were the single leg countermovement jump (SL-CMJ) and the single leg hop lateral (SL-HL). The magnitude of asymmetry in the SL-CMJ test (10.80%) was higher compared to that of the SL-LH test (6.47%), and there were no significant differences between particular categories. The direction of asymmetry represented by the Kappa values showed “poor” and “fair” levels of agreement in U14 and U16 categories, which means that asymmetries rarely favored the same side during each jumping test, but in contrast, in the U18 category the Kappa value result was “slight”. There were significant correlations between SL-CMJ jumping asymmetries and jumping performance in the U18 category (r = 0.31 to 0.49). These data indicate that in order to identify the player's asymmetry profile, we need to consider the magnitude and the direction of different tests which will help better understand the natural deficits, contextualize them and consider appropriate training interventions for the reduction of inter-limb asymmetries.

## Introduction

Handball is a sport characterized by the continuous repetition of high-intensity tasks, especially on one leg, such as jumps and changes of direction (Manchado et al., 2013; Michalsik and Aagaard, 2015). Repetition and the preference of one lower limb over the other when performing these movements in such a specific sporting context causes neuromuscular adaptations that lead to the development of inter-limb asymmetries in the lower limbs (Dos’Santos et al., 2020).

The difference in strength, coordination, and motor control between limbs is a potential risk factor for injury to the anterior cruciate ligament (ACL), especially among adolescent girls (Hewett et al., 2005; Myklebust et al., 1998). After maturation, the absence of adequate strength and reductions in motor control lead to rapid physiological and anthropometric changes (Myer et al., 2004), resulting in altered movement strategies and ultimately, limb dominance (Maloney, 2019). Consequently, limb dominance and asymmetry have been measured across numerous physical capacities such as muscle strength (Beato et al., 2019; Bishop et al., 2019; Newton et al., 2006), balance (Atkins et al., 2016; Fort-Vanmeerhaeghe et al., 2019), change of direction speed (Arboix-Alió et al., 2021; Fílter et al., 2021; Madruga-Parera et al., 2019a, 2020a), and unilateral jumping (Lockie et al., 2014; Madruga-Parera et al., 2020b).

In jumping assessments, which are relevant to handball (Madruga-Parera et al., 2020a), the magnitude of asymmetry has been observed as being specific to the type of the jump being tested. Lockie et al. (2014) and Arboix-Alió et al. (2021), respectively, reported asymmetries of 10.4% and 9.8% in the single leg countermovement jump (SL-CMJ), 3.3% and 3.6% in the single leg hop (SL-H), and 5.1% and 3.3% in the single leg lateral hop (SL-LH). This notion of task-specificity also applies to the direction of the imbalance (i.e., the superior performing limb). Madruga-Parera et al. (2020b) showed that the dominant limb in one type of the jump (SL-CMJ, SL-H, and SL-LH) was rarely dominant in another test, in 42 youth handball athletes (Kappa = −0.05 to 0.15). Bishop et al. (2020) achieved similar results in a study of 18 adolescent female soccer players in whom differences between limbs rarely favoured the same limb in three unilateral jump tests, a SL-CMJ, a drop jump (DJ) and a squat jump (SJ) (Kappa = 0.02 to 0.45). This information highlights the need to utilize more than a single test to measure asymmetry and to ensure that the direction of asymmetry (i.e., limb dominance) is considered in any subsequent analysis.

Despite the volume of literature surrounding asymmetry in recent years, few studies have taken into account the interaction between different chronological ages or maturational status. Read et al. (2017) showed that landing force asymmetry during the SL-CMJ was significantly greater for adolescent soccer players in maturational stages close to the peak growth rate (circa-PHV) and post-PHV compared with those who were in the pre-PHV phase (d = 0.41–0.43; *p* < 0.001). In contrast, Madruga-Parera et al. (2019b) described that the highest asymmetry values in the SL-CMJ (19.31% ± 12.19) were in the circa-PHV or U14 maturational stage in adolescent tennis players, but with no significant differences between chronological or maturational ages. Similarly, Pardos-Mainer et al. (2021) found no significant differences between U14, U16 and U18 adolescent soccer players, when measuring lower limb asymmetry values in the SL-CMJ and SL-H and speed during a change of direction task. To the authors’ knowledge though, these three studies represent the only ones to compare asymmetry across different maturational groups and with little consensus between them, further research is warranted in this regard. Further to this, identifying both the magnitude and the direction of imbalances during jump tasks (which is highly relevant for handball) (Karcher and Buchheit, 2014), may provide an opportunity to improve performance of any limb showing a clear and consistent deficit compared to the other (Maloney, 2019), noting that two of these aforementioned studies did not factor in the direction of asymmetry into their analyses (Pardos-Mainer et al., 2021; Read et al., 2017).

Therefore, the objectives of this study were to: 1) evaluate the magnitude and the direction of jump asymmetries (vertical and lateral) in three different age groups of adolescent female handball players, and 2) identify the effect of asymmetry between limbs on jump performance in the three age groups. It was hypothesized that adolescent female handball players would show greater asymmetry in U14 and U16 age groups due to biological development, but that it would be in the U18 age group where the direction of asymmetry would favour the same limb when comparing the two tests due to training and sport specialization. Regarding the second aim, it was hypothesized that this asymmetry would affect performance in jump tasks.

## Methods

### Participants

One hundred and eighty-five adolescent female handball players participated voluntarily in this study (age: 14.88 ± 1.49 years; body height: 161.62 ± 6.67 cm; body mass: 57.9 ± 9.4 PHV: 2.08 ± 0.98). The sample was classified, according to their chronological age and the category in which they competed, into U14 (n = 57; 31%), U16 (n = 87; 47%), and U18 (n = 41; 22%). Biological age was calculated using a regression equation that included measures of age, body mass, standing height, and sitting height (Mirwald et al., 2002). The maturational phase of each player was determined at the time of data collection: early maturation (pre-PHV), defined as one year before PHV; average maturation (circa-PHV), ± 1 year after PHV; and late maturation, more than 1 year after PHV (post-PHV) (Sherar et al., 2005). Most of the players were classified in a late maturation phase (158/185; 85%) and most of them were right-handed (173/185; 94%), so they used their left leg to shoot (Table 1). All study participants trained and competed with their respective teams in the top tier league for their category. They all attended three training sessions per week, each lasting approximately 120 minutes, and one match per week. None of them performed complementary strength training outside the on-court sessions. Exclusion criteria were: a) having previously had an ACL injury, b) not having attended 80% of the sessions during the month prior to the tests, and c) presenting any type of injury (overload or acute) on the day of the tests. The sample size was determined using G*Power3 software for Mac (Faul et al., 2007). In order to evaluate the differences between the three independent groups with a statistical power (1-β) of 0.8, with an alpha level of 0.05 and an effect size of 0.8, a total of 159 players were required. All participants and their respective legal guardians were informed of the procedures, methods, benefits, and possible risks of participating in the study prior to giving their written consent (guardians). In addition, the investigation was carried out with the consent of the managers of the handball clubs to which the players belonged. This study was approved by the ethics committee of the General Secretariat for Sport of Catalonia (20/2019/CEICEGC).

**Table 1:** Descriptive data of participants (mean ± standard deviation).

	Total (n = 185)	U14 (n = 57)	U16 (n = 87)	U18 (n = 41)
Chronological age (years)	14.88 ± 1.49	13.18 ± 0.59	15.01 ± 0.54	16.99 ± 0.64
Peak height velocity (PHV)^a^	2.08 ± 0.98	1.01 ± 0.61	2.22 ± 0.44	3.29 ± 0.52
Pre-PHV^b^	1 (0.5%)	1 (2%)		
Circa-PHV^b^	26 (14.05%)	26 (45%)		
Post-PHV^b^	158 (85.40%)	30 (53%)	87 (100%)	41 (100%)
Experience (years)	6 ± 2	5 ± 2	6 ± 2	8 ± 2
Body mass (kg)	57.9 ± 9.4	54 ± 9.5	58.9 ± 8.1	61 ± 10.2
Height (cm)	161.62 ± 6.67	158.96 ± 7.47	162.52 ± 5.56	163.41 ± 6.7
BMI (kg/m^2^)	22.1 ± 2.89	21.29 ± 2.99	22.3 ± 2.7	22.9 ± 2.97
Throwing arm (L/R)	12/173	1/56	5/82	6/35

Note. ^a^ Estimated biological age (Mirwald et al., 2002). ^b^ Number and percentage of participants. L: left throwing arm; R: right throwing arm.

### Measures

#### Single Leg Countermovement Jump (SL-CMJ)

To calculate unilateral vertical jump height, a contact platform (Chronojump Bosco system, Barcelona) was used measuring in centimetres based on the flight time calculation method (Bosco et al., 1983). Each player was instructed to stand in the centre of the platform on the designated leg, hands on hips and with the non-jumping leg bent at ~90° to avoid counter-lateral propulsion (Read et al., 2017). When the player was ready, they had to perform a countermovement to a self-selected depth before jumping as high as possible (Madruga-Parera et al., 2019a). They were also instructed to land on both feet simultaneously to increase the possibility of a stable landing (Meylan et al., 2009). The attempt was not taken into account if there was any visible contribution from the non-jumping leg, if the jumping leg was not fully extended during flight, or if the hands left the hips.

#### Single Leg Lateral Hop (SL-LH)

A measuring tape fixed to the ground was used to calculate lateral jump distance on one leg. Each participant was instructed to stand on the designated leg with their foot just behind the start line (0 cm) with their hands on their hips. When the player was ready, they had to perform a countermovement to a self-selected depth before jumping laterally as far as possible. For example, when the player started with their left leg, the jump had to be as far to the right as possible with hands fixed to hips throughout the jump. The landing had to be performed simultaneously with both feet, shoulder-width apart, and the landing had to be stable for three seconds (Madruga-Parera et al., 2019a). The distance was measured from the outer edge of the foot closer to the start line to 0 cm.

### Design and Procedures

A cross-sectional study was conducted to determine performance in unilateral jump tasks and to quantify the magnitude and the direction of asymmetry between limbs in adolescent female handball players. Vertical unilateral and lateral jumps were assessed using a SL-CMJ and a SL-LH, respectively. The tests were conducted on a single day during the third month of the competition period (November). Previously, all participants had conducted multiple familiarization sessions during training to avoid any learning effects being present during the tests. Before warming up, height, seated height, and body mass of each participant were recorded. Players then completed a 10-min warm-up based on the RAMP (raise, activate, mobilize, potentiate) system (Jeffreys, 2019). In the potentiation phase, three jumps were performed on each leg and for each test at a perceived intensity of 50, 75 and 100% of the maximal effort. Upon completion of the warm up, three minutes of rest were allowed before data collection began. The starting leg was randomized in each test and three successful attempts were recorded for each leg and jump. Between each attempt there was a 60-s rest period. The best jump on each leg was used for subsequent analysis.

### Statistical Analysis

The mean and standard deviation (SD) were calculated for all variables. The assumptions of normality were verified by the Kolmogorov-Smirnov test. The reliability of the intra-session measurements was analysed using the coefficient of variation (CV) and the type-2 intra-class correlation coefficient (ICC) with absolute agreement and 95% confidence intervals. CV values were considered acceptable when CV ≤ 10% (Turner et al., 2015) and the interpretation of ICC values was: ICC < 0.50 = poor, 0.50–0.74 = moderate, 0.75–0.90 = good and > 0.90 = excellent (Koo and Li, 2016).

The magnitude of asymmetry between limbs was quantified as the percentage difference between the two limbs (left and right) using the following equation: ((100/(maximum value) * (minimum value) * − 1 + 100) * IF (left < right, 1, −1) (Bishop et al., 2018a). The IF function at the end of the formula determines the direction of asymmetry, without compromising the magnitude. To evaluate the consistency of dominance between limbs using a common metric in two tasks, the Kappa coefficient was calculated and interpreted as: poor (≤ 0), slight (0.01–0.20), fair (0.21–0.40), moderate (0.41–0.60), substantial (0.61–0.80), almost perfect (0.81–0.99) and perfect (1) (Viera and Garrett, 2005).

The Wilcoxon test was used to detect differences in performance of each test between dominant (DOM) and non-dominant (NDOM) limbs for the entire sample. Analysis of the differences in asymmetry values and the differences in performance values on DOM and NDOM limbs between age groups, was done using analysis of variance (one-way ANOVA), the Levene test was performed to examine the homogeneity of variance. If the results of the Levene’s test were statistically significant, it was corrected using Welch method. Magnitude of difference between groups was also computed through Cohen’s *d* effect sizes. These were interpreted in line with Hopkins et al. (2009) where < 0.20 = trivial; 0.20–0.60 = small; 0.61–1.20 = moderate; 1.21–2.0 = large and > 2.0 = very large. The relationship between asymmetry values and performance values for each test was assessed using a Spearman bivariate correlation analysis. Data were processed anonymously using a code system. The significance level was set at *p* < 0.05 for all tests. Analyses were performed using the SPSS statistical program for Mac (version 27, IBM, New York) and JASP for Mac (version 14.1; JASP Team (2020), University of Amsterdam, The Netherlands).

## Results

The consistency between attempts for each age group of the SL-LH test was acceptable, presenting CV values below 10%, while slightly elevated CV values (>10%) were present for the SL-CMJL and SL-CMJR. All ICC values were considered moderate and good (between 0.66 and 0.87) (Table 2).

**Table 2:** Coefficient of variation (CV) and interclass correlation coefficient (ICC) with 95% confidence intervals for each age group (n = 185).

	U14 (n = 57)		U16 (n = 87)		U18 (n = 41)	
	CV (%)	ICC (95%CI)	CV (%)	ICC (95%CI)	CV (%)	ICC (95%CI)
SL-CMJ_L_	12.78	0.68 (0.57; 0.78)	9.65	0.81 (0.75; 0.87)	10.36	0.67 (0.52; 0.79)
SL-CMJ_R_	10.72	0.75 (0.64; 0.83)	10.20	0.80 (0.73; 0.86)	8.61	0.66 (0.50; 0.79)
SL-LH_L_	6.82	0.85 (0.74; 0.91)	6.13	0.80 (0.71; 0.87)	4.97	0.79 (0.67; 0.87)
SL-LH_R_	6.93	0.82 (0.71; 0.90)	5.17	0.87 (0.78; 0.92)	6.29	0.69 (0.48; 0.79)

Note. SL-CMJ_L_: single leg countermovement jump with the left leg; SL-CMJR: single leg countermovement jump with the right leg; SL-LH_L_: single leg lateral hop with the left leg; SL-LH_R_: single leg lateral hop with the right leg.

The highest magnitudes of asymmetry were observed in the SL-CMJ test. No significant differences were found between asymmetry values by categories (Table 3). Eleven players obtained a value of zero in the magnitude of the asymmetry of the SL-LH test. The results of these players were ruled out from the Kappa index analysis as the direction of asymmetry could not be defined.

**Table 3:** Mean data ± standard deviation (SD) and the index of asymmetry for each age group and effects sizes between groups.

	U14 (n = 57)	U16 (n = 87)	U18 (n = 41)	Effect size, U14 vs. U16	Effect size, U14 vs. U18	Effect size, U16 vs. U18
SL-CMJ_L_ (cm)	13.33 ± 2.96	13.25 ± 3.05	13.62 ± 2.71	0.03	−0.10	−0.13
SL-CMJ_R_ (cm)	12.49 ± 2.78	12.84 ± 2.99	13.15 ± 2.12	−0.31	0.35	−0.03
Asy SL-CMJ (%)	11.30 ± 9.10	10.14 ± 7.11	11.50 ± 7.15	0.15	−0.03	−0.19
SL-LH_L_ (cm)	112.53 ± 20	118.13 ± 16.76	118.61 ± 12.81	−0.31	−0.35	−0.03
SL-LH_R_ (cm)	113.25 ± 17.39	115.84 ± 16.52	117.71 ± 11.25	−0.15	−0.39	−0.12
Asy SL-LH (%)	6.85 ± 5.62	6.27 ± 4.50	6.38 ± 4.89	0.12	0.09	−0.02

Note. SL-CMJ_L_: single leg countermovement jump with the left leg; SL-CMJ_R_: single leg countermovement jump with the right leg; Asy SL-CMJ: asymmetry between legs for the single leg countermovement jump test; SL-LH_L_: single leg lateral hop with the left leg; SL-LH_R_: single leg lateral hop with the right leg; Asy SL-LH: asymmetry between legs for the single leg lateral hop test.

**Figure 1:**
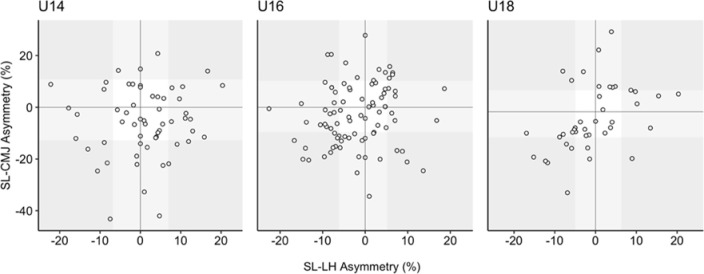
Asymmetry profile for each player and test (SL-CMJ and SL-LH) in the U14, U16 and U18 categories. Positive values indicate right leg dominance and negative values indicate left leg dominance. The shaded areas correspond to the threshold of the percentage of the CV of each of the tests, shown in Table 2. Grey-white: asymmetry percentage lower than the CV threshold in both tests; light grey background: asymmetry percentage lower than the CV in at least one of the two tests, and dark grey background: higher asymmetry percentage than the CV in both tests.

The Kappa coefficients comparing the consistency of the direction of asymmetry between the SL-CMJ test and the SL-LH test showed that asymmetry favoured the same side between tests “poorly” in the U14 category (Kappa = −0.03), “regularly” in the U16 category (Kappa = 0.29), but they did so “moderately” in the U18 category (Kappa = 0.59). The asymmetry profile of each player for the SL-CMJ test and the SL-LH test is shown in Figure 1. The boundaries of the shaded areas correspond to the CV of each of the tests (Table 2), bearing in mind that asymmetry can only be significant if the percentage value is greater than the test point of variability (Bishop, 2021; Bishop et al., 2018b; Exell et al., 2012). Players with an asymmetry value higher than the test CV are in the outermost and darkest areas.

The Wilcoxon test showed that there was a significant difference between the performance values of the DOM and the NDOM leg in SL-CMJ tests (DOM: 13.85; NDOM: 12.31) and SL-HL (DOM: 199.90; NDOM: 112.07) (W = 17205; *p* < 0.001 and W = 15225; *p* < 0.001) with a large sample effect (rB = 1), but there were no significant differences between performance values of the DOM leg between age groups in the SL-CMJ (F = 0.47; *p* = 0.62) and the SL-LH (F = 1.45; *p* = 0.24) test. There were also no significant differences with the NDOM leg in the SL-CMJ (F = 0.42; *p* = 0.66) and the SL-LH test (F = 1.81; *p* = 0.17).

## Discussion

The aims of this study were, firstly, to establish an asymmetry profile in jumping tasks based on the SL-CMJ and the SL-LH in adolescent female handball players of different age groups, and secondly, to determine the relationship between these asymmetries and measures of physical performance. The results showed no significant differences in the magnitude of asymmetry by age groups. Significant associations were found when examining the effects of asymmetry on one-footed jump performance. The greatest asymmetries in the SL-CMJ test were associated with lower NDOM leg performance in the same test in U16 and U14 categories, and the greatest asymmetries in the SL-LH test were also associated with lower performance by the NDOM leg of the same test in the U18 category. In contrast, there was a positive correlation between asymmetry in the SL-CMJ jump and performance in the DOM leg jump in both tests, and in the NDOM leg in the SL-HL jump in the U18 category. Furthermore, the direction of asymmetry rarely favoured the same side between tests in the younger categories.

The magnitude of asymmetry in the SL-CMJ test (10.80%) was higher compared to that of the SL-LH test (6.47%). These results are consistent with previous studies in handball which described asymmetries of 8–11% for the SL-CMJ and 5–8% for the SL-LH in male adolescent players (Madruga-Parera et al., 2019a, 2020a) and of 9% for the SL-CMJ and 3% for the SL-LH in female adolescent players (Arboix-Alió et al., 2021). In fact, these data are in line with research from other sports as well. Lockie et al. (2014) reported asymmetries of ~10% for the SL-CMJ and ~5% for the SL-LH in 30 adult team sport players, and Raya-González et al. (2021) reported asymmetries of ~14% for the SL-CMJ and ~5% for the SL-LH in 16 adolescent female soccer players. What is really significant is the fact that the values of asymmetry between limbs differed between the test having enlarged the sample (n = 185). It is therefore clear that the magnitude may vary depending on the task used to quantify imbalance, and robustness is provided by demonstrating that it is difficult to use a single threshold that may indicate associations with lower performance or a possible increased risk of injury (Bishop, 2021). Furthermore, given this task-specificity associated with asymmetry (Madruga-Parera et al., 2019a), it is our suggestion that more than one test is required to determine an athlete’s inter-limb asymmetry profile (Bishop, 2020).

There were no significant differences between age groups in the asymmetries of the SL-CMJ and SL-LH (Table 3). Pardos-Mainer et al. (2021) did not find any significant differences either in the same age groups in a sample of 54 adolescent female soccer players, and neither did Madruga-Parera et al. (2019b) in 41 adolescent male tennis players classified into categories from U12 to U18. In the present study, the results indicate that asymmetries are independent of the maturational phase in which they find themselves, as the total number of players who belonged to these two categories (n = 128) was in a late stage of maturation (post-PHV) (Table 1). To explain this point further, and one that is often glossed over in asymmetry research, the standard deviation in relation to the mean was very large (Table 3), indicating a huge amount of within-group variability of movement among this young population (Read at al., 2019). This further highlights the need for more than a single test to analyse asymmetry and where possible, to undertake individual data analysis (Bishop et al., 2018a), although this can be challenging to compute in the present study, given the large sample size.

For the direction of asymmetry, results showed between “poor” and “fair” levels of agreement (Kappa = −0.03 to 0.29) in the U14 and U16 categories, respectively. Arboix-Alió et al. (2021) also detected “slight” levels of agreement (Kappa = 0.10) for consistency in the same jump tests and in a similar sample (adolescent female handball and basketball players), and Pardos-Mainer et al. (2021) described “fair” and “poor” levels in U14 and U18 categories, respectively, but in this case between the SL-CMJ test and the SL-H test. These data indicate that limb dominance rarely favours the same limb when comparing it between tests (Virgile and Bishop, 2020). To somewhat contrast these findings, the U18 category displayed a “moderate” level of agreement between the two jump tests, whereby only eight players (8/41 = 19.51%) exhibited fluctuations in limb dominance characteristics between tests (Figure 1). This slightly better level of agreement in the U18 category shows that these two tests share some similarities in limb dominance (Karcher and Martin, 2012; Ortega-Becerra et al., 2020). However, the lower Kappa values in the younger age groups could be a result of reduced levels of coordination (due to maturation status), resulting in greater fluctuations in performance variability, as opposed to pure limb capacity deficits.

Another point to consider was that there were significant differences between the performance of the DOM limb and the NDOM limb in the two tests and among the three categories (Table 4). These results could be explained by the specific demands of the sport where the handball player uses, during practice, one limb more frequently than the other. Taking into account that the jumping action is performed with the leg opposite to the throwing arm, the repetition of the specific skill will allow a better development of the strength capacity in the jumping actions. Madruga-Parera et al. (2019a) found the same results, but in COD actions.

**Table 4:** Dominant (DOM) and non-dominant (NDOM) leg performance for each test and according to the competitive category.

	U14 (n = 57)	U16 (n = 87)	U18 (n = 41)	Effect size, U14 vs. U16	Effect size, U14 vs. U18	Effect size, U16 vs. U18
SL-CMJ DOM	13.72 ± 2.97	13.75 ± 3.06	14.24 ± 2.54	−0.01	−0.19	−0.17
SL-CMJ NDOM	12.10 ± 2.59	12.34 ± 2.81	12.53 ± 2.01	−0.09	−0.18	−0.07
SL-LH DOM	116.97 ± 18.66	120.81 ± 16.42	122.05 ± 10.85	−0.22	−0.32	−0.08
SL-LH NDOM	108.81 ± 17.91	113.16 ± 16.04	114.27 ± 11.93	−0.26	−0.35	−0.08

Note. SL-CMJ DOM: single leg countermovement jump with the dominant leg; SL-CMJ NDOM: single leg countermovement jump with the non-dominant leg; SL-LH DOM: single leg lateral hop with the dominant leg; SL-LH NDOM: single leg lateral hop with the non-dominant leg.

Finally, there was a significant correlation between SL-CMJ asymmetries and improvement in dominant leg performance in both jumps (SL-CMJ and SL-LH) at the U18 level (Table 5). These results may be due to the accumulation of training volume as the player progresses through the ranks considering the one-sidedness of handball-specific actions (Maloney, 2019). In contrast, the correlation was significantly negative in U14 and U16 categories only for NDOM vertical leg jump performance. These latter results were consistent with those of Fort-Vanmeerhaeghe (2019) in 81 adolescent boys and girls handball, basketball and football players, and with those of Madruga-Parera et al. (2020b) in adolescent male handball players. Although this asymmetry in many cases can be considered a risk factor for injury (Bishop et al., 2018b), we must also bear in mind that the demands of sport, as we have indicated above, can lead to the development of functional asymmetries and these can be beneficial for the improvement of performance of the conducted tests.

**Table 5:** Spearman correlation coefficient between test performance and asymmetries.

Test	SL-CMJ asymmetry			SL-HL asymmetry		
	U14	U16	U18	U14	U16	U18
SL-CMJ DOM	0.13	0.13	0.49**	0.04	0.09	−0.11
SL-CMJ NDOM	−0.28*	−0.22*	0.10	0.10	0.07	−0.16
SL-HL DOM	−0.10	0.03	0.36*	0.18	0.16	−0.04
SL-HL NDOM	−0.04	0.03	0.31*	−0.19	−0.17	−0.46**

Note. * the correlation is significant at the 0.05 level (bilateral); ** the correlation is significant at the 0.01 level (bilateral). SL-CMJ DOM: vertical countermovement jump with the dominant leg; SL-CMJ NDOM: vertical countermovement jump with the non-dominant leg; SL-HL DOM: lateral countermovement jump with the dominant leg; SL-HL NDOM: lateral countermovement jump with the non-dominant leg.

The three main limitations of the study were, first, that the sample was not classified according to maturational age. Study participants belonged to teams organized according to their chronological age. Only one player in the pre-PHV phase was identified in the U14 category of competition, as female players reach PHV before males (Lloyd and Oliver, 2012). Future studies should collect equitable samples in the three maturational phases described by Sherar et al. (2005), to compare how biological age influences the asymmetry profile and to determine whether there is a relationship between performance and asymmetry between maturational phases. Second, the data were collected cross-sectionally at a specific point during the season and this prevented determining any causal relationship between particular variables. In addition, only a one-leg vertical and lateral countermovement jump was investigated. Future studies should include a third direction (horizontal) in order to define more precisely the asymmetry profile in jump tasks of adolescent female handball players. Finally, the sample size for each category was not the same, which may have influenced the significance of the results.

## Conclusions

The SL-CMJ and SL-LH tests show asymmetries in jump actions in adolescent female handball players. The greatest asymmetries are observed in the SL-CMJ test, and there are no significant differences between the three age groups (U14, U16 and U18). These asymmetries influence jumping performance, but only the U18 category showed significant values when relating a low asymmetry to an increase in performance. The direction of the asymmetry rarely favours the same leg when compared between the two tests in the U14 and U16 categories, but moderately so in the U18 category. The data from this study suggest that each adolescent female handball player has an individual and unique profile. This profile depends on the magnitude and the direction of that asymmetry, which is also test-specific.

Although these results show no differences of asymmetry levels among the different groups, it is possible to outline certain differences (asymmetry-performance relationship and asymmetry direction) between the U-18 group and the two younger groups. According to this point, we suggest that athletes perform neuromuscular training adapted to their maturational age to follow up the variation of inter-limb asymmetries through the different biological stages, but it is also important to consider the possible relationship between asymmetry magnitude and performance in athletes at the end of their junior period. This differentiation should be taken into account when planning tests and training and when analyzing the obtained results as well.
